# A Retrospective Audit of Pharmacologic and Non-Pharmacologic Management of Childhood Acute Asthma Exacerbation at Usmanu Danfodiyo University Teaching Hospital, Sokoto: Adherence to Global Treatment Guidelines

**DOI:** 10.3389/fphar.2020.531894

**Published:** 2020-08-31

**Authors:** Kazeem Adeola Oshikoya, Ibrahim Abayomi Ogunyinka, Shallom Ese Imuzei, Bilkisu Ilah Garba, Nma Mohammed Jiya

**Affiliations:** ^1^Department of Pharmacology, Therapeutics and Toxicology, Lagos State University College of Medicine, Ikeja, Nigeria; ^2^Department of Clinical Pharmacy and Pharmacy Practice, Usmanu Danfodiyo University, Sokoto, Nigeria; ^3^Department of Pediatrics and Child Health, Usmanu Danfodiyo University, Sokoto, Nigeria

**Keywords:** asthma, children, exacerbation, triggers, non-pharmacologic, pharmacologic, guidelines, treatment

## Abstract

**Background:**

Adequate management of childhood acute asthma exacerbation requires optimal non-pharmacotherapy and pharmacotherapy. Global asthma guidelines provide critical information and serves as a quick reference decision-support material for clinicians.

**Objectives:**

We aimed at evaluating hospital management of childhood acute asthma exacerbation to ascertain its conformity to the global treatment guidelines, and to identify factors that predict short or prolonged observation in the hospital.

**Method:**

This was a retrospective audit of the management of acute asthma exacerbation in children seen between 01 January 2017 and 31 December 2018 at Usmanu Danfodiyo University Teaching Hospital (UDUTH), Sokoto, Nigeria. Relevant data on demography, asthma triggers and severity, functional and clinical diagnoses, types of controller medications used before and after presentation, non-pharmacotherapy and pharmacotherapy instituted during presentation, duration of observation in the hospital, and treatment outcomes were extracted from the case file of each eligible patient.

**Results:**

A total of 119 children presented with features of suspected acute asthma exacerbations during the study period but only 63 (52.9%) that met the inclusion criteria for the study were included for analysis. The 63 children that were evaluated had mild (47; 74.6%) and moderate (16; 25.4%) acute asthma exacerbations. Their median (interquartile range) age was 8 (5–15) years. More males (36; 57.1%) than females (27; 42.9%) presented with features of the condition. Majority (50; 79.8%) of the patients had at least one trigger factor and of the 73 trigger factors reported, cold weather (19; 26.0%) was the commonest. Nebulized salbutamol (48; 76.5%), in addition to intravenous (23; 57.9%) and oral (17; 42.5%) corticosteroids, was used during hospital treatment. Patients were discharged mostly on short course of oral corticosteroid only (37; 58.8%). Of the 17 major recommendations in the Global Initiative for Asthma (GINA) guidelines, good (5; 29.4%), moderate (7; 41.2%), and poor (5; 29.4%) levels of adherence were observed. Specifically, moderate and poor levels of adherence were observed in the management of 61(96.8%) and 2(3.2%) patients, respectively. The odds of admission for ≤12 h were higher for female children and patients with mild cases.

**Conclusion:**

Good and moderate adherence levels to 12 of the 17 GINA recommendations were observed in our center. Nonetheless, reinforcement of institutional guidelines for acute asthma management is suggested to further improve the quality of care of childhood acute asthma exacerbations.

## Introduction

Global Initiative for Asthma (GINA) defined asthma as a heterogeneous disease, usually characterized by chronic inflammatory disease of the airways, which is associated with widespread but variable outflow obstruction (Global Initiative for Asthma (GINA), 2016). Asthma manifests clinically as wheeze, recurrent cough, breathlessness, and chest tightness (Global Initiative for Asthma (GINA), 2016). Recent evidence from Northern Nigeria suggests that prevalence of childhood asthma is 0.2%–1.0% in the community (Ozoh et al., 2019) and 3.2%–12.5% from hospital records (Akhiwu et al., 2017; Ibraheem et al., 2020) compared to 18.4% previously reported by the International Study of Asthma and Allergies in Childhood (International Study of Asthma and Allergies in Childhood (ISAAC) Steering Committee, 1998). In spite of this improved prevalence, control of childhood asthma remains a health challenge in Nigeria and other low-income countries. In fact, physicians’ non-adherence to the existing treatment guidelines for asthma is a major contributory factor to poor treatment outcomes (Gupta and Weiss, 2009). Treatment guidelines, otherwise called clinical practice guidelines, are documents systematically developed from evidence-based medicine to guide decisions and criteria regarding diagnosis, management, and treatment in specific areas of health care (Gopalakrishnan et al., 2014). The use of asthma treatment guidelines could reduce childhood asthma visits and hospitalizations to the pediatric emergency department, thus saving an estimated $1.3 to $1.59 billion annually in the United States (Gupta and Weiss, 2009; Perry et al., 2019), and saving a cumulative national cost of $0.16 billion constituting 0.002% of the national GDP in Nigeria (Ughasoro et al., 2020). Ensuring that asthma treatment guidelines become an integrated and useful part of health care in low-and middle-income countries, however, remains a challenge (Zar and Lalloo, 2013).

Despite free online access to the GINA guidelines for asthma management and several other asthma guidelines, which usually adapt to specific country needs, the burden and prevalence of childhood asthma remain significantly high globally (Ferrante and La Grutta, 2018). Poor adherences of physicians to the available institutional or international treatment guidelines have impacted the clinical outcomes of childhood asthma negatively (Canonica et al., 2007). There is evidence of poor knowledge of the GINA guidelines for asthma management among pediatric residents and physicians in other specialties in Nigeria (Ayuk et al., 2017a). Lack of understanding of the content of the guidelines (Adeniyi et al., 2017) and poor adherence (Ayuk et al., 2017a; Adeniyi et al., 2017) are also common among Nigerian doctors. Further, public and private hospital physicians had knowledge gaps in managing childhood asthma (Chima et al., 2017).

The treatment goals of asthma are to reduce the underlying inflammation and prevent recurring attacks and clinical symptoms (Global Initiative for Asthma (GINA), 2016). However, the treatment goals may not be fully achieved at home due to poor adherence to the recommended guidelines for patients (Murphy et al., 2012). In-patients with moderate to severe acute asthma exacerbations or those who respond poorly to bronchodilator therapy, before presenting to a hospital, require inhaled bronchodilators and systemic corticosteroids as a major component of their treatment; however, some of them may not respond sufficiently well to avoid admission (Global Initiative for Asthma (GINA), 2016). Managing such patients may be problematic in Nigeria due to knowledge gaps in standard practice (Adeniyi et al., 2017; Ayuk et al., 2017a; Chima et al., 2017). Medications used to manage acute asthma exacerbations differ from those used in chronic stable states. Given the role of airway inflammation in childhood asthma, anti-inflammatory medicines now form the mainstay of treatment. It is, therefore, necessary to improve the quality of care by ensuring that physicians treating children with asthma adhere to the available treatment guidelines, especially in the use of anti-inflammatory agents.

Notwithstanding the non-pharmacologic measures recommended in the GINA guidelines for acute severe asthma management, pharmacotherapy remains the cornerstone of treatment in children and adults, especially during acute exacerbations. The GINA is a widely accepted guidelines defining the principles of pharmacologic and non-pharmacologic treatments of asthma and indicate age specific treatments in steps, according to clinical severity and level of disease control (Global Initiative for Asthma (GINA), 2016). A global asthma physician and patient (GAPP) survey reported that, despite inhaled corticosteroids (ICS) being the first-line therapy for asthma (Chapman et al., 2017), physicians tend to under-prescribe these drugs and may even prescribe long acting β_2_-agonists (LABAs) for mild persistent asthma for which the GINA guidelines recommended ICS monotherapy (Global Initiative for Asthma (GINA), 2016).

Earlier studies from Nigeria evaluating clinical management of asthma in children and adults according to the GINA and other international guidelines were questionnaire based and focused on physicians’ knowledge of GINA guidelines, application of the guidelines’ contents in clinical practice, and availability of facilities and resources in tertiary hospitals to support international standards for asthma management (Desalu et al., 2016; Adeniyi et al., 2017; Ayuk et al., 2017a; Chima et al., 2017). However, the physicians’ claim of good knowledge of and adherence to the treatment guidelines may not necessarily translate into appropriate implementation of the guidelines in clinical practice. A recent global asthma physician survey showed that physicians generally did not use standardized tools to monitor asthma control or to manage its treatment and, despite high awareness of single maintenance and reliever therapy (SMART), the strategy were often commonly misapplied (Chapman et al., 2017). An audit of adherence to the GINA guidelines during childhood acute asthma exacerbations is very necessary to improve treatment and to achieve better outcomes. We, therefore, audited the quality of acute asthma care among children that presented to a tertiary hospital in Northwestern Nigeria in compliance with the GINA guidelines. We also determined factors that predicted duration of observation for acute asthma exacerbations in children.

## Methods

### Design and Setting

This was a retrospective study involving 119 children aged 1–15 years with features of suspected acute asthma exacerbations; however, only the case files of 63 children ≥5 years and ≤ 15 years old were reviewed according to the study protocol. These children were treated for acute asthma exacerbations at the Emergency Pediatric Unit of Usmanu Danfodiyo University Teaching Hospital (EPU-UDUTH), Sokoto in Northwestern Nigeria, between 1^st^ January 2017 and 31^st^ December 2018. The hospital serves as a referral center to primary, secondary and tertiary hospitals in Northwestern Nigeria. This hospital also provides an internship and residency training programs in all pediatric specialties. The hospital is staffed with a consultant pulmonologist, resident physicians, and several other internists involved in the running of a pulmonary clinic. Several consultants in other specialties are also involved in asthma management upon presentation to the EPU. Available infrastructures for asthma management include pulse Oximeters, peak flow meters, nebulizer, oxygen concentrator, and intensive care units (ICUs) for management of severe acute cases.

Children ≥ 5 years with asthma presenting with an acute exacerbation (old and new cases) were those previously receiving care in primary healthcare centers, private hospitals in the state, family medicine clinic within the hospital, or the pulmonary out-patient clinic. The patients with acute asthma exacerbations are seen at the EPU and first attended to by a house officer on duty, and later reviewed by a resident doctor. The attending house officer evaluates and institutes therapy before the resident doctor on call reviews the patient. During a review, the treatment may be modified; the patient may be discharged upon improvement; or admitted to the ward or ICU, depending on asthma severity, to be further evaluated and managed by the managing consultant. All patients discharged from the EPU, ward or ICU are given1-2-week appointment for follow up at Pulmonology clinic.

### Patient Selection

**Figure 1:**
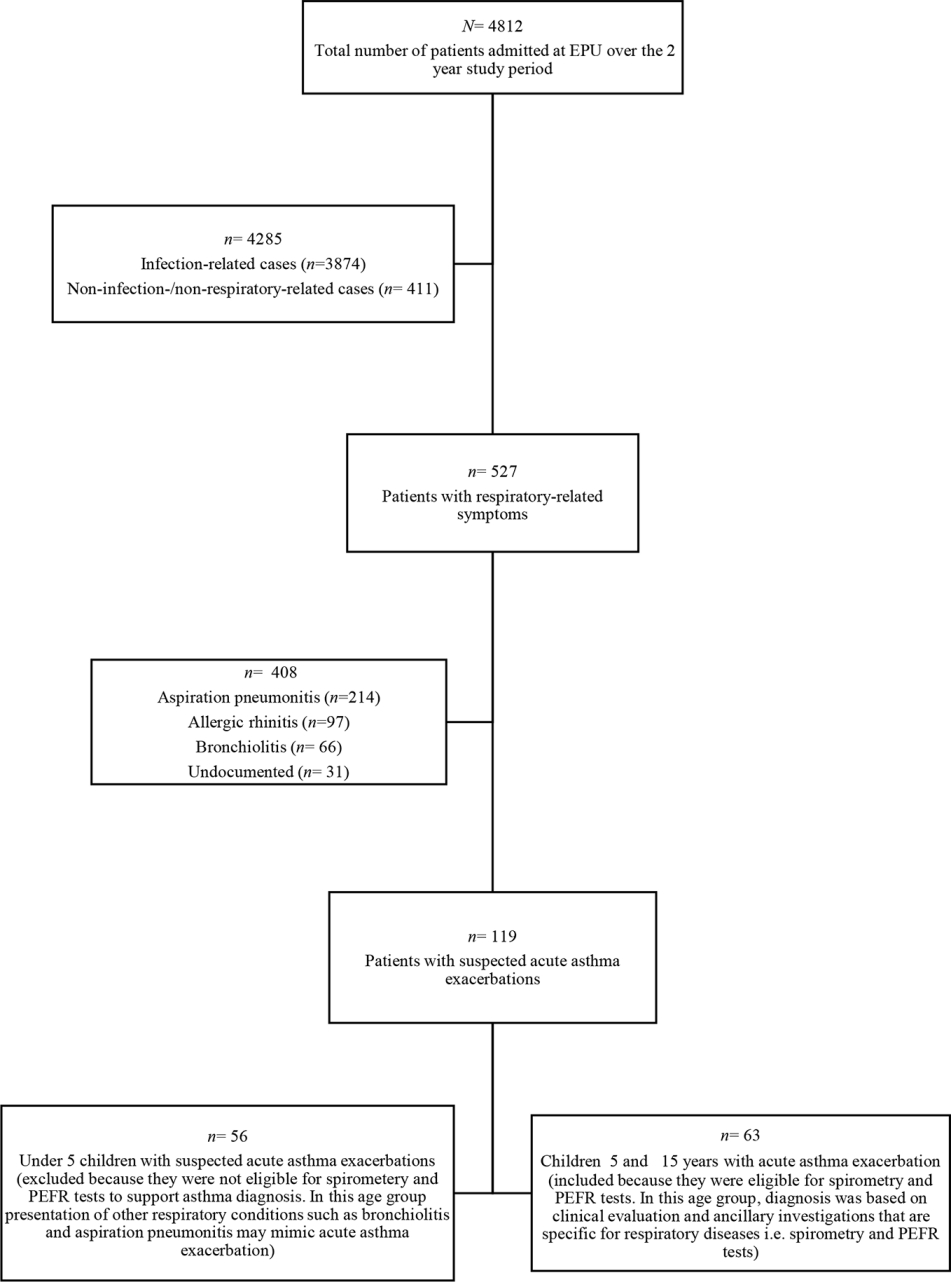
Flow chart indicating patient selection process at EPU between 1^st^ January, 2017 and 31^st^ December, 2018 at Usmanu Danfodiyo University Teaching Hospital, Sokoto. PEFR refers to Peak Expiratory Flow Rate; EPU refers to Emergency Pediatric Unit.

We used the admission register at the EPU to identify 119 cases of suspected acute asthma exacerbations admitted over the 24-month study period. The registration numbers were then used to retrieve the case files from the health record’s office. We reviewed the case file of each patient to identify the eligible ones and included those that met the inclusion criteria such as aged between 5 and 15 years old, documentation of complete information, documented evidence of diagnosed acute asthma exacerbations, patients who received at least a single anti-asthma medication and not discharged immediately after receiving treatment. This resulted in 63 eligible case files that constituted the sample size eventually analyzed in the study (**Figure 1**). We collected the necessary information using an audit *pro forma* developed and validated by the British Thoracic Society (2015), which was used for similar study among pediatric asthmatic patients in Sudan (Ibrahim et al., 2012). The information extracted includes socio-demographic details, clinical profile of the patient such as clinical features, time, month and season of admission, comorbid conditions related to asthma, emergent investigations, anti-asthma medications, duration of observation in the hospital, and the treatment outcome. We further categorized the duration of observation as short, if ≤ 12 h or long, if >12 h. Treatment was categorized as standard if compliant with the GINA 2016 recommendations (Global Initiative for Asthma (GINA), 2016).

### Protocol for Managing Childhood Acute Asthma Exacerbation at UDUTH (Adapted from the GINA Guidelines)

The Pulmonology unit has a standard protocol displayed conspicuously on the wall of EPU for all doctors in the pediatric department to follow when managing emergency cases of childhood acute asthma exacerbations. The protocol is adapted from the GINA guidelines and focuses on both pharmacologic and non-pharmacologic therapies, recognizing the features of mild, moderate, severe and life-threatening asthma. Important recommendations were to administer oxygen for moderate to severe cases and to maintain SpO_2_ levels >95%, to administer nebulized or inhaled short acting inhaled β_2_-agonists (SABA) (salbutamol which is readily available) every 20 min for an hour and intravenous or oral corticosteroids. Inadequate response after an hour of therapy warrants the additional use of nebulized ipratropium bromide every 20 min for an hour, repeated after 4 h. If response persistently remains poor, add intravenous magnesium sulfate as an infusion, a loading dose of SABA followed by infusions, or administer a loading dose of aminophylline followed by infusions. Identify and/or treat the trigger factors and develop an avoidance plan, provide patients with a written asthma management plan for home treatment, and give a short follow up appointment for patient to be seen at a Pulmonology/allergy clinic.

We evaluated adherence to the GINA guidelines in relation to important recommendations indicated on the EPU-UDUTH protocol for asthma management, functional assessments of asthma using a spirometer, peak expiratory flow meter in children ≥ 5 years old, and oxygen saturation measurement with an Oximetry and repeat measurements every 1–2 h after initiating treatments.

### Asthma Diagnosis and Severity

Asthma was established in children ≥5 years old based on a history of variable respiratory symptoms such as wheeze, breathlessness, chest tightness, recurrent cough, and airflow limitation that is responsive immediately to bronchodilators or ICS within a week. Presence of a family history of asthma and/or other allergic conditions, such as allergic rhinitis or eczema, in addition to the respiratory symptoms, further reinforces the diagnosis of asthma (Global Initiative for Asthma (GINA), 2016).

We excluded children <5 years old because an assessment of acute asthma in early childhood could be difficult as the seemingly episodic wheeze and cough that characterized the condition is also common in children without asthma (Pedersen, 2007; Brand et al., 2014) and routine assessment of airflow limitation is quite challenging in this age group (Global Initiative for Asthma (GINA), 2016). Further, intermittent wheezing attacks are usually due to viral infections and the response to asthma medication is inconsistent. Asthma exacerbations (asthma attacks or acute asthma) were defined as episodes of progressive increase in breathlessness, wheeze, recurrent cough, and chest tightness, or presence of a combination of these symptoms in association with decreased lung function, as determined by PEFR or ratio of forced expiratory volume in 1 second (FEV_1_) to forced vital capacity (FVC) that is less than 0.9 in children with a normal status (Global Initiative for Asthma (GINA), 2016).

There are no asthma severity scoring tools validated for use in the pediatric in-patient setting; therefore, asthma severity was classified as mild, moderate, severe or life-threatening as presented in **Table 1**, modified from the Consensus Guidelines for Inpatient Management of Asthma: Northern California Pediatric Hospital Medicine Consortium (Northern California Pediatric Hospital Medicine Consortium, 2015) and Expert Panel Report 3 National Asthma Guidelines (National Heart, Lung, and Blood Institute and National Asthma Education and Prevention Program, 2007).

**Table 1:** Asthma severity rating*.

Symptoms and signs	Mild	Moderate	Severe	Life threatening (impending respiratory arrest)
Respiratory rate	<3 mo: <56 3–6 mo: <50 6–12 mo: <44 1–4 yr: <38 5–8 yr: <31 >9 yr: <25	<3 mo: 56–68 3–6 mo: 51–60 6–12 mo: 45–53 1–4 yr: 39–45 5–8 yr: 32–38 >9 yr: 26–30	<3 mo: > 69 3–6 mo: >61 6–12 mo: >54 1–4 yr: >53 5–8 yr: >39 >9 yr: >31	Normal and/or slowing due to inability to maintain work of breathing
Prolonged expiration	Normal to minimally prolonged	Prolonged	Prolonged	Variable
Auscultation	None or end expiratory wheezes only	Throughout exhalation	Inspiratory/expiratory wheeze OR absent due to poor air exchange	Diminished/absent due to poor air exchange
Retractions	None or minimal intercostal retractions	Intercostal and sub-sternal retractions +/- nasal flaring	Grunting OR Tripoding OR intercostal, sub-sternal and supraclavicular retractions	Tiring, inability to maintain work of breathing
Dyspnea	With activity or agitation	While at rest *Infants*: soft or shorter cry, difficulty feeding	While at rest *Infants*: Stop feeding	While at rest
Initial PEFR (or FEV_1_)	PEF ≥70% predicted or personal best	PEF 40%−69% predicted or personal best	PEF <40% predicted or personal best	PEF <25% predicted or personal best

PEFR refers to Peak Expiratory Flow Rate.

FEV_1_ refers to Forced Expiratory Volume in 1 Min.

mo refers to age in months.

yr refers to age in years.

*indicates asthma severity for inpatients was adapted from Northern California Pediatric Hospital Medicine Consortium (2015) and National Heart, Lung, and Blood Institute and National Asthma Education and Prevention Program (2007).

### Adherence to the GINA Guidelines Using a Scoring Method

Previous studies evaluating physicians’ adherence to asthma treatment guidelines did not use scoring methods (Zar and Lalloo, 2013; Desalu et al., 2016; Ayuk et al., 2017a). Data from such studies should; therefore, be interpreted with caution. However, there are reports of use of physicians’ adherence scores for patients with chronic heart failure in a multicenter study where the adherence score is defined as a ratio of the treatment actually prescribed to the treatment that should theoretically have been prescribed (Komajda et al., 2017). This method was adopted in our study as we defined adherence score to asthma treatment guidelines as a ratio of the evaluation and treatment actually implemented by physicians to the evaluation and treatment that should theoretically have been implemented. The theoretical treatment score was calculated for every patient, considering the pharmacologic and non-pharmacologic treatments recommended in the guidelines.

The adherence score was calculated for each patient by summing the points attributed as follows: 0 points for non-evaluation or non-implementation of the pharmacologic treatment. 0.5 points for non-evaluation of the non-pharmacologic recommendations due to lack of necessary equipment in the hospital (assuming ≥ 50% of the doctors would have used the equipment if available). 1 point for evaluating or implementing the non-pharmacologic recommendations, and for implementing the pharmacologic recommendations. Further, administration of recommended drugs, oxygen in moderate to severe cases or non-administration of oxygen in mild cases during presentation to the hospital was scored 1 point. The score ranged from 0 (very poor) to 1 (excellent) and we defined three levels of adherence: good adherence (score =1); moderate adherence (score >0.5 to<1); and poor adherence (score ≤0.5). In this study, our term ‘adherence’ relates solely to physicians following guidelines, not to patient compliance.

### Ethical Approval

The health and research ethics committee of UDUTH approved the study with a reference number UDUTH/HREC/2018/No. 750.

### Data Analysis

The demographic and clinical characteristics for three adherence groups- good (score =1), moderate (score >0.5 to <1) and poor (score ≤0.5) are presented using descriptive statistics as numbers and percentages for categorical variables, and means ± standard deviation or median for continuous variables. The values for each characteristic were compared between groups using an analysis of variance (ANOVA) test for quantitative variables and chi-square test or Fisher’s exact test for qualitative variables. Multivariate logistic regression was performed with duration of observation as the primary outcome and those parameters with significant *p*-values in the univariate analysis as covariates. Data were analyzed using SPSS statistics software, version 21.0. Armonk, NY, USA: IBM. Corp (Released 2012). All *p*-values < 0.05 were considered statistically significant, and all statistical tests were two-tailed.

## Results

### Demographics and Asthma Profile for the Patients

A total of 119 children presented with features of suspected acute asthma exacerbations during the study period but only 63(52.9%) that met the inclusion criteria were evaluated in this study (**Figure 1**). The median (interquartile range [IQR]) age of the patients was 8(5-15) years. More males (36; 57.1%) than females (27; 42.9%) presented with acute asthma exacerbations. The patients presented with mild (47; 74.6%) and moderate (16; 25.4%) asthma exacerbations. There was no statistically significant association between gender of the patients and asthma severity (*p*=0.996, Fisher’s exact test). About one-third (24; 34.9%) of the patients had at least one asthma related allergic condition, of which 22 (91.7%) had rhinitis only and 2 (8.3%) had both rhinitis and allergic conjunctivitis.

### Diagnostic and Ancillary Investigations for Asthma

Over one-half (33; 52.4%) of the patients had PEFR performed on them. Neither FEV_1_ nor FVC was conducted on all the patients due to lack of a spirometer. Peripheral oxygen saturation (SpO_2_) levels on admission and, its serial measurements, while on treatment, were performed for all the 16 (25.4%) patients with moderate asthma exacerbations.

### Environmental and Physical Trigger Factors

**Figure 2:**
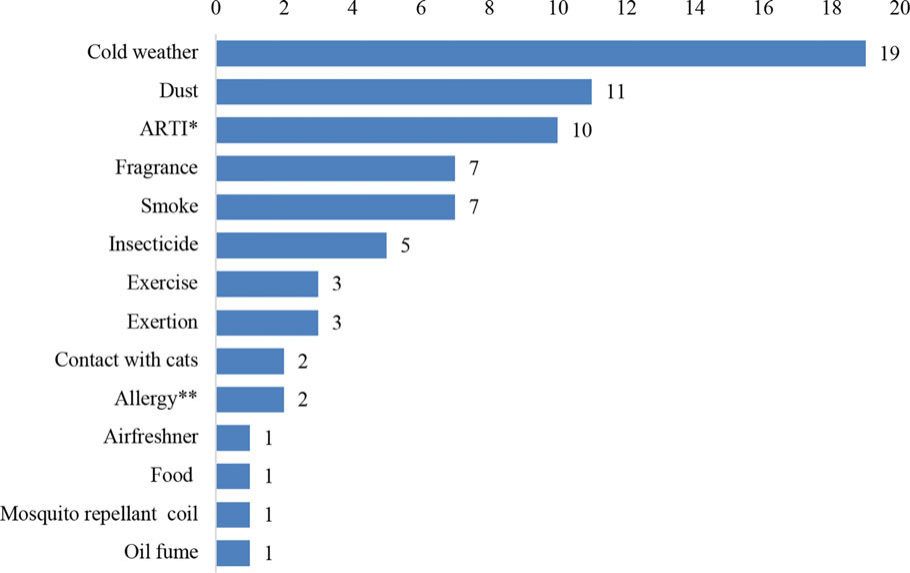
The trigger factors reported among 50 children presenting with acute asthma exacerbation. ARTI* refers to preceding acute repiratory tract infection. Allergy** refers to preceding allergic rhinitis, conjunctitis or dermal atopy.

Family history of allergy or asthma was documented for all the patients. Majority (50; 79.8%) of the patients had at least one trigger factor. Of the 73 trigger factors reported (**Figure 2**), cold weather (19; 26.0%) was the most common.

### Adherence to Controller Medications Prior to Presentation

**Figure 3:**
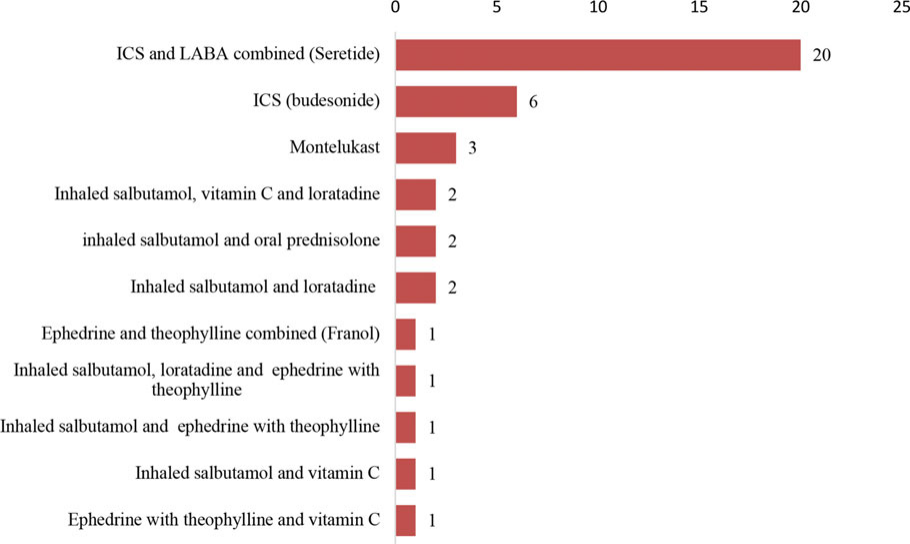
Medicines used regularly by 40 patients on controller medicines prior to observation in the hospital. ICS refers to inhaled corticosteroid. LABA refers to long-acting β_2_-agonist. Seretide^®^ refers to a combination of fluticasone and salmeterol. Franol^®^ refers to a combination of ephedrine and theophylline.

Only 40 (63.0%) patients were regularly or interruptedly using controller medications prior to the onset of acute exacerbations of their asthma. ICS (fluticasone) combined with LABA (salmeterol) was the controller medicine used by most of the patients (20; 50.0%) prior to presentation (**Figure 3**). Inhaled salbutamol was the reliever medicine used by 30(48.0%) patients prior to presentation.

### Treatment of Acute Asthma Exacerbations During Admission

**Figure 4:**
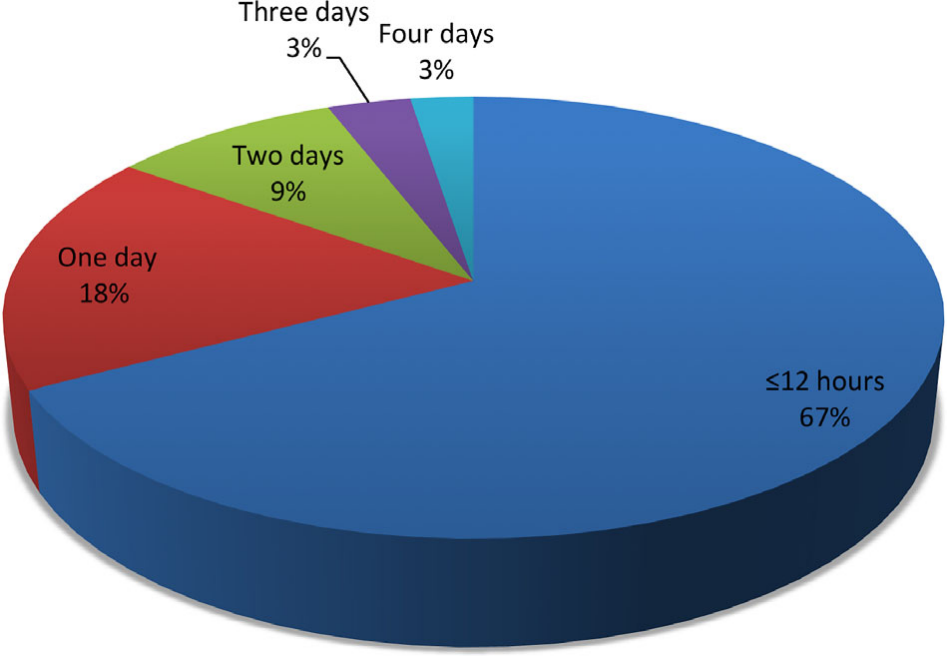
Duration of admission for children with acute asthma exacerbation.

Patients were observed in the hospital for a short duration of ≤12 h or >12 h (1–4 days) during acute asthma exacerbations (**Figure 4**). Majority (47; 73.9%) of the patients were observed in the hospital for ≤ 12 h. Nebulized salbutamol was the initial bronchodilator used to treat acute asthma exacerbations in 48(76.5%) patients. Additional intravenous aminophylline was received by 9(58.1%) of the 16 patients with moderate asthma exacerbations. Systemic corticosteroids (40; 63.9%), administered as intravenous (23; 57.5%) or oral (17; 42.5%), were used to treat airway respiration among the patients.

All patients with moderate asthma exacerbations received supplemental intranasal oxygen, based on measured SpO_2_ levels, while 14 (22.7%) had oral (11; 81.5%) or intravenous (3; 18.5%) antibiotics comprising amoxicillin (5; 33.3%), ampicillin/cloxacillin (4; 29.6%), cefuroxime (3; 25.9%), amoxicillin/clavulanate (1; 7.1%), and erythromycin (1; 7.1%).

### Take Home Controller Medications and Provision of Written Asthma Action Plan After Hospital Discharge

All the patients were discharged on medications comprising oral corticosteroids only (37; 58.8%), a combination of inhaled bronchodilator (long-acting β_2_-agonist- salmeterol) and corticosteroids (fluticasone) (12; 19.1%), inhaled bronchodilator (short-acting β_2_-agonist) only (9; 15.1%), inhaled corticosteroids (budesonide) only (4; 6.7%), or a combination of inhaled bronchodilator (short-acting β_2_-agonist) and oral corticosteroids (1; 2.5%).

Written asthma action plan was made available to the parents of all the patients and specific follow-up appointment (1-2 weeks) was documented for 24 (38.8%) patients after discharge home.

### Documentation of Inhaler Technique Review With Patient or Their Parents on Discharge

There was no documentation of the knowledge of the patients or their parents regarding appropriate use of the inhaler technique prior to admission. However, the inhaler technique was reviewed with all the 63(100.0%) patients and their parents prior to discharge from admission.

### Adherence to the GINA Treatment Guidelines

In **Table 2**, we assessed the adherence scores for and the level of adherence to 17 major recommendations in the GINA guidelines. Of the 17 recommendations, good (5; 29.4%), moderate (7; 41.2%), and poor (5; 29.4%) levels of adherence were observed. Further, evaluation of the total adherence level to all the 17 major recommendations for each patient treated by the doctors revealed moderate and poor levels of adherence in the management of 61(96.8%) and 2(3.2%) patients, respectively. Good adherence level to all the 17 recommendations was not observed.

**Table 2:** Adherence to Global Initiative for Asthma (GINA) guidelines during hospital admission of 63 children with acute asthma exacerbation.

GINA Recommendation	Adherence score	Adherence level
Assessment of asthma attack based on history and physical examination	0.84	Moderate
Documentation of child or parents’ smoking status	1.00	Good
Documentation of trigger factors for asthma	0.79	Moderate
Documentation of adherence to controller medicines at home	0.64	Moderate
Functional assessment of asthma severity on admission		
*PEFR*	0.52	Moderate
*FEV_1_*	0.50	Low
*FVC*	0.50	Low
*FEV_1_/FVC*	0.50	Low
Peripheral oxygen saturation (SpO_2_) measured	1.00	Good
Administration of supplemental oxygen (O_2_)	1.00	Good
Administration of nebulized salbutamol (SABA)	0.74	Moderate
Administration of systemic (oral or intravenous) corticosteroid within an hour of hospital admission	0.64	Moderate
Asthma management action plan for patient	1.00	Good
Use of ICS and LABA by patient after discharge	0.19	Low
Use of oral corticosteroid by patient after discharge	0.59	Moderate
Inhaler technique review with patient or their parents on discharge	1.00	Good
Short follow up appointment after discharge	0.38	Low

GINA refers to global initiative for asthma.

PEFR refers to peak expiratory flow rate.

FEV_1_ refers to forced expiratory volume in a minute.

FVC refers to forced vital capacity.

ICS refers to inhaled corticosteroid.

SABA refers to short-acting β_2_-agonist.

LABA refers to long-acting β_2_-agonist.

### Predictors of Duration of Observation of Patients With Acute Asthma Exacerbations

The demographics, clinical variables, pharmacologic and non-pharmacologic parameters for patients with short (≤ 12 h) and long (>12 h) durations of observation were compared in a univariate analysis (**Table 3**). There were significant associations between duration of observation of the patients and their gender (*p*=0.036), asthma severity (*p*=0.009), and administration of intranasal oxygen (*p*=0.009).

**Table 3:** Univariate analysis of the factors associated with duration of admission for 63 children with acute asthma exacerbation.

**Factors**	**Duration of admission**		***p*-value**
	**≤12 h** ***n*(%)**	**>12 h** ***n*(%)**	
Gender			**0.036**
*Male*	35(97.2)	1(2.8)	
*Female*	21(77.8)	6(22.2)	
Presence of asthma related comorbidity			0.699
*Yes*	22(91.7)	2(8.3)	
*No*	34(87.2)	5(12.8)	
Season of admission			0.999
*Rainy*	14(93.3)	1(6.7)	
*Dry*	42(87.5)	6(12.6)	
Adherence to controller medicines at home			0.678*
*Antiasthma medicines*	25(92.3)	2(7.7)	
*Non-antiasthma medicines*	18(85.7)	3(14.3)	
*No medicine used*	13(86.7)	2(13.3)	
Asthma severity			**0.009**
*Mild*	45(95.7)	2(4.3)	
*Moderate*	11(68.7)	5(31.3)	
Presence of trigger factors			0.382
*Yes*	44(93.6)	5(6.4)	
*No*	12(85.7)	2(14.3)	
Use of controller medicines prior to admission			0.699
*Yes*	36(90.0)	4(10.0)	
*No*	20(86.9)	3(13.1)	
Asthma evaluation with PEFR			0.429
*Yes*	28(84.8)	5(15.2)	
*No*	28(93.3)	2(6.7)	
Administration of nebulized SABA during observation in the hospital			0.999
*Yes*	42(87.5)	6(12.5)	
*No*	14(93.3)	1(6.4)	
Administration of antibiotics during admission for observation			0.259
*Yes*	7(77.8)	2(22.2)	
*No*	49(96.1)	5(3.9)	
Administration of systemic (intravenous or oral) corticosteroids within an hour of admission for observation			0.233
*Yes*	39(92.8)	3(7.2)	
*No*	19(82.6)	4(17.4)	
Administration of intranasal oxygen			**0.009**
*Yes*	11(68.7)	5(31.3)	
*No*	45(95.7)	2(4.3)	
Short follow up appointment (1-2 weeks)			0.236
*Yes*	23(95.8)	1(4.2)	
*No*	33(84.6)	6(15.4)	

PEFR refers to peak expiratory flow rate.

SABA refers to Short-acting β_2_-agonist.

*Refers to p-value based on Pearson Chi-Square test.

Other p-values are based on Fisher’s exact test.

The bold p-values show that they are statistically significant.

In multivariate analysis of pharmacologic and non-pharmacologic variables associated with duration of observation of the patients, adjusting for the three covariates with significant *p*-values in the univariate analysis, only gender and asthma severity were significantly associated with long (>12 h) duration of observation in the hospital. The odds of long observation in the hospital were lower for male compared to female children (adjusted odds ratio: 0.07, 95%CI: 0.01–0.73). Also, the odds of long observation in the hospital were lower for children with mild compared to moderate asthma exacerbation (adjusted odds ratio: 0.07, 95%CI: 0.01–0.49) (**Table 4**).

**Table 4:** Multivariate analysis of the factors associated with duration of admission for acute asthma exacerbation.

Factors	*P*-value	Odds ratio (95% confidence interval) for short observation in the hospital^a^	
		Unadjusted^b^	Adjusted^c^
Gender	0.026	**0.10(0.11-0.89)**	**0.07(0.01-0.73)**
Asthma severity	0.008	**0.09(0.02-0.57)**	**0.07(0.01-0.49)**
Administration of intranasal oxygen	0.842	2.93(0.95-9.06)	1.16(0.28-4.83)

SABA refers to Short-acting β_2_-agonist.

a The model was generated using binary logistic regression.

b Unadjusted odds ratio (OR) refers to the OR for each variable with no others in the model.

c Adjusted OR refers to the OR after all variables were included in the model.

Odds ratio with statistically significant p value is in bold.

## Discussion

Good total adherence to all the 17 GINA recommendations was not observed in this study. Rather, we observed mostly moderate total adherence level in 96.8% of the patients. However, specific assessment of adherence to each recommendation for all the patients showed a good adherence to 5 of the 17 recommendations (documentation of child or parents’ smoking status, measurement of peripheral oxygen saturation (SpO_2_) levels, administration of supplemental intranasal oxygen (O_2_), provision of asthma management action plan for patient, and review of inhaler technique with patient or their parents before discharged home), which are generally non-pharmacologic managements. Regarding pharmacologic management of the patients, adherence to administration of nebulized SABA, administration of systemic (oral or intravenous) corticosteroids within an hour of hospital admission, and prescription of oral corticosteroids to the patient after discharge was moderate, while it was poor to prescription of ICS and LABA to the patients.

This is the first study to assess adherence to asthma treatment guidelines using a scoring method. Earlier studies assessing doctors’ adherence to asthma treatment guidelines were subjective as the adherence levels were not objectively measured. Therefore, their findings should be interpreted with caution. Larger studies are, however, required to validate our scoring method. Notwithstanding, our findings on total adherence are contrasting to the low adherence reported in previous pediatric (Adeniyi et al., 2017) and adult (Desalu et al., 2016) studies in Nigeria. Low adherence to GINA guidelines had been reported among 59.1% resident doctors managing childhood acute asthma exacerbations in a tertiary hospital in Southeastern Nigeria (Ayuk et al., 2017a). The improvement in adherence observed in our study may be attributed to the dedication of the pediatric team at UDUTH and due to enforcement of the protocol for managing childhood acute asthma exacerbations as adapted from the GINA guidelines for use at our emergency pediatric unit.

In the initial assessment of childhood acute asthma exacerbations, the GINA guidelines recommended good history and physical examination, knowing the smoking status of the child and/or the parent, presence of trigger factors, adherence to controller medications at home, and functional assessment of asthma severity. However, adherence was good to only documentation of presence of trigger factors and moderate to other recommendations for initial asthma assessment. Desalu et al. (2016) reported poor documentation of history/physical examinations and smoking status of adult asthma patients in Nigeria compared to moderate adherence to their documentation in our study. A previous study involving 30 children on emergency admission for acute asthma exacerbations in Lagos, Nigeria showed that trigger factors were documented for 26.7% of the patients (Okoromah et al., 2006), which was far lower than the observed 79.8% in our patients. Similarly, a high rate (71.8%) for trigger factors had been documented among adult asthma patients in Nigeria (Desalu et al., 2016).

We observed moderate adherence to current medications and functional indices of acute asthma severity, using PEFR in our patients. Poor adherence levels to both recommendations had been reported in previous pediatric and adult studies in Nigeria (Okoromah et al., 2006; Desalu et al., 2016). Poor adherence to current medications is a known risk for acute asthma exacerbations in children and adults (Engelkes et al., 2015), thus emphasizing the need for documenting history of current antiasthma medications.

Pulmonary function tests (FEV_1_, FVC, and FEV_1_/FVC), as measured by spirometers, are more sensitive than clinical symptoms or physical examination in making diagnoses of obstructive respiratory diseases including asthma (Moore, 2007). Unfortunately, they were not performed on our patients during presentation and treatment monitoring due to lack of spirometers in the pediatric department. However, we recorded moderate adherence to these recommendations based on an assumption that, if the equipment needed to perform the assessment were available, ≥50% of the doctors would use them effectively. Thus, we may have over- or under-scored adherence to these recommendations. Lack of objective tests to support the diagnosis of asthma has contributed to inappropriate over diagnosis, under diagnosis and misdiagnosis of asthma among school children in Canada (Yang et al., 2017). Ayuk et al. (2017b) in their review observed that spirometry is currently under-used in both tertiary and primary care settings in resource poor countries, which was attributed to lack of institutional guidelines for asthma management, lack of spirometers and, where available, healthcare workers were improperly trained to use the tools and interpret the findings appropriately.

None of the patients presented with severe asthma exacerbations. This finding is surprising given the high rates reported in local and international studies (Edelu et al., 2016; Dondi et al., 2017). A plausible explanation for this finding is that nearly all the patients were registered old patients of UDUTH; the only tertiary public hospital in the entire Sokoto State that runs a pediatric Pulmonology clinic. At the clinic, majority of the parents have been well educated on how to identify the signs and symptoms of severe asthma exacerbations and the necessary measures needed to take to avert progression to a severe form before presenting to the EPU. Another possibility is that parents might have taken their children with severe acute asthma exacerbations to traditional herbal medicine practitioners for treatment since alternative and complementary therapy use is on the increase among children with asthma (Philp et al., 2012; Javid et al., 2019). However, nearly a three-quarter of the patients had mild asthma, which is similar to the pattern reported among pediatric population in Nigeria (Oviawe and Osarogiagbon, 2013; Kuti and Omole, 2017). This finding may be attributed to the rapid recognition of the signs and symptoms of acute asthma exacerbations by the parents or caregivers and their willingness to access hospital in time. Avoidance of exposure of the children to the trigger factors is also possible since a larger number of the patients had one or more identified trigger factors. The spectrum of trigger factors observed in our study are similar to those previously reported among pediatric population in Nigeria (Kuti and Omole, 2017) but differ from those reported in Italy (Dondi et al., 2017). Cold weather, followed by dust and ARTI, were the commonest triggers in our patients, while ARTI, followed by exercise, dust and cold weather were the predominant triggers reported by Kuti and Omole (2017). By contrast, respiratory tract infections, followed by allergy, were the major triggers in the Italian study (Dondi et al., 2017).

Regarding non-pharmacologic management of our patients, SpO_2_ levels were measured in those patients with moderate exacerbations only. Based on experience, SpO_2_ levels were rarely <95% in children presenting to the EPU with mild asthma exacerbation and thus routine measurement of SpO_2_ levels was rarely performed for this group of patients. Further, children with mild exacerbations were often observed for ≤12 h after presentation and they rarely require oxygen supplementation. Desalu et al. (2016) reported SpO_2_ levels measurement and administration of supplemental O_2_ in 9.9% and 30.7% of adult patients, respectively. Higher proportion of adult patients in Spain (Linares et al., 2006) and pediatric patients in Italy (Dondi et al., 2017) received supplemental O_2_, which was guided by the SpO_2_ values.

Nearly a three-quarter of our patients were admitted for ≤12 h probably due to most cases being mild asthma exacerbation. This is similar to the 74.6% of mild to moderate cases admitted for such short duration in another study from Nigeria (Edelu et al., 2016). The short duration of admission by most of the patients may be a pointer to the efficiency of appropriate management plan instituted in all cases. Appropriate management is evident by the use of nebulized salbutamol (SABA) in over a three-quarter of the patients, use of systemic (intravenous or oral) corticosteroids in nearly two-third of the patients, and use of aminophylline for less than 5.0% of the patients within an hour of admission. Okoromah et al. (2006) reported the administration of nebulized salbutamol and oral corticosteroids to 80.0% of their patients during emergency admission, in addition to frequent use of aminophylline. Another study in Sudan reported the administration of nebulized salbutamol and systemic corticosteroids, respectively, to 99.2% and 74.6% of their patients during emergency admission, in addition to the use of aminophylline by less than 1.0% of the patients (Ibrahim et al., 2012). Our findings were, however, similar to the moderate adherence to administration of nebulized salbutamol (Desalu et al., 2016) and systemic corticosteroids within an hour of admission (Onyedum et al., 2014; Desalu et al., 2016) among adult patients in Nigeria and Spain (Linares et al., 2006). Nebulized SABAs, at low dose, are the recommended first line treatment for acute asthma in children of all ages with fewer adverse effects than oral or intravenous use (Global Initiative for Asthma (GINA), 2016). The rare use of aminophylline by our patients is in line with the GINA guidelines. This is due to the narrow therapeutic index and high toxicity of aminophylline, which precludes its use in children, unless ICS are unavailable (Global Initiative for Asthma (GINA), 2016).

The GINA guidelines have recommended that, following discharge from admission, controller medications for children should be commenced, which include inhaled corticosteroids (ICS), a combination of ICS and long-acting β _2_-agonists (ICS/LABA), leukotriene receptor antagonists (LTRA) and Cromones (Global Initiative for Asthma (GINA), 2016). On discharge, 58.8% of our patients were prescribed oral corticosteroids only, while 19.1% were prescribed ICS/LABA combination (Seretide^®^). This is an improved result compared to the previous ones from Nigeria where Iloh et al. (2017) reported low prescription rate of corticosteroids among physicians managing childhood asthma or even over a decade ago where most children with asthma were not prescribed any medication upon discharge from admission (Okoromah et al., 2006). Although it has been established that most adult patients in Nigeria use their inhalers incorrectly (Onyedum et al., 2014), all our patients on inhalers had their inhaler techniques reviewed. This is in contrast to previous adult studies in Nigeria (Desalu et al., 2016) and United Kingdom (Lindsay et al., 2012), where 35.5%–40.8% of the patients had their inhaler techniques reviewed on discharge. All our patients were given asthma action plan compared to the 6.7% and 9.9% given similar instructions among pediatric and adult asthma patients, respectively, in Nigeria (Okoromah et al., 2006; Desalu et al., 2016).

Most of the patients were given specific follow-up appointment; however, only 38.8% were reviewed within 1–2 weeks of discharge. This low rate may be attributed to the fact that parents felt the child was better; hence their reluctance to come back for follow up. However, the low rate of follow-up observed here is similar to the 31.7% reported among adult asthma patients in Nigeria (Desalu et al., 2016) but lower than the 66.8% reported in the United Kingdom (Lindsay et al., 2012). Lack of follow-up is a common problem among pediatric asthma patients in Nigeria (Okoromah et al., 2006) and in the United States (Smith et al., 2006; Zorc et al., 2009). These comparative studies had reported low rates of follow-up visits in their patients after discharge from emergency admissions. Regular follow-up visits are useful to help build a doctor and patient relationship, to predict medication adherence, and to ensure adequate monitoring of the pulmonary function of the patients.

Previous studies had not predicted the factors associated with short and long admission duration among children with acute asthma exacerbation. However, our study suggested that the odds of observation in the hospital for >12 h were lower for male compared to female children. The odds of long observation in the hospital were also lower for children with mild compared to moderate asthma exacerbations. Various studies with varying methodologies, sample sizes, and different settings had reported factors predicting hospitalization for asthma exacerbations in children (Lyell et al., 2005; Buyuktiryaki et al., 2013; Alherbish et al., 2018). In a larger multicenter study in Turkey, severity of asthma exacerbations in children was a major predictive factor for hospitalization (Buyuktiryaki et al., 2013). Contrary to our findings, male gender and administration of systemic steroid in the first hour predicted short period of hospitalization in the Turkish study. However, the odds of near fatal asthma pediatric intensive care unit admission of male children were seven times higher than those of female (Lyell et al., 2005). The small sample size, a single center study, dearth of pediatric pulmonologist to appropriately supervise the residents and interns in our center, which characterized our study may have affected the effects of the covariates on our predicting model. It is hoped that these problems would be addressed in a larger study in the future.

Inaccuracies from documentations in the medical records of patients, irretrievable medical record files, and lack of information regarding functional assessment to support the diagnosis of asthma severity are major limitations that could relatively undermine our results. We were also aware that individuals treated for respiratory diseases such as bronchiolitis, allergic rhinitis, and aspiration pneumonitis may have presented with signs and symptoms mimicking acute severe asthma (Sharma and Gupta, 2019), as well as received some doses of anti-asthma medications such as corticosteroids, thus over-estimating the proportion of asthma cases reported here. Further, exclusion of under 5-year-old children may have resulted in under-estimation of the true prevalence of asthma in our study. However, exclusion of this group of children was necessary due to the challenges of making an accurate diagnosis; since asthma presentation can mimic other respiratory diseases in young children. We applied a scoring method to determine adherence level to the GINA guidelines. Although the method had been previously used in several studies assessing adherence to antihypertensive and anti-heart failure drugs in adults (Komajda et al., 2017), its validation in children with asthma is hereby advocated. Although information regarding training of the patients or their parents on the inhaler techniques was documented for all the patients prior to hospital discharge, no information was documented for their understanding of the processes before and after admission to help guide future care. Given the moderate adherence to the GINA recommendation for PEFR evaluation in childhood asthma, we assumed over 50% of the doctors would have performed FEV1, FVC, and FEV1/FVC, if the needed equipment were available. Therefore, a score of 0.5 awarded for these parameters may have under- or over-estimated the adherence levels for the three parameters. Considering this is a single center study involving a small sample size, we exercised caution in generalizing our findings to the entire country. It is, however, hoped that this study would serve as a template for future national childhood asthma study to improve adherence to global guidelines.

## Conclusion

Good and moderate adherence levels to 12 of the 17 GINA recommendations were observed among doctors managing acute asthma exacerbations in children at the study center, while non-use of a combination of ICS and LABA by patients after discharge and default from short follow up appointment after discharge were the most violated. When all the 17 recommendations were specifically evaluated for each patient, a moderate total adherence level was observed in the management of nearly all the patients. The odds of long admission duration (>12 h) were lower in male compared to female patients. The odds were also lower for children with mild compared to moderate exacerbation. Reinforcing the use of institutional asthma treatment guidelines or the global guidelines is necessary to further improve treatment of asthma exacerbation in children.

## Data Availability Statement

All datasets generated for this study are included in the article/supplementary material.

## Ethics Statement

The health and research ethics committee of UDUTH approved the study.

## Author Contributions

KO conceptualized and designed the study. He also analyzed the data and developed the first draft of the manuscript. IO took active part in conceptualizing the study. He was involved in data acquisition and entry, and reviewed the initial draft of the manuscript for critical intellectual content. SI was involved in conducting literature review for the study, data acquisition, and reviewed the initial draft of the manuscript. BG and NJ supervised data collection and reviewed the draft manuscript before final approval. All authors contributed to the article and approved the submitted version.

## Conflict of Interest

The authors declare that the research was conducted in the absence of any commercial or financial relationships that could be construed as a potential conflict of interest.
